# Dysfunctional GABAergic inhibition in the prefrontal cortex leading to "psychotic" hyperactivation

**DOI:** 10.1186/1471-2202-9-41

**Published:** 2008-04-25

**Authors:** Shoji Tanaka

**Affiliations:** 1 Department of Information and Communication Sciences, Sophia University, 7-1 Kioicho, Chiyodaku, Tokyo, 102-8554, Japan

## Abstract

**Background:**

The GABAergic system in the brain seems to be dysfunctional in various psychiatric disorders. Many studies have suggested so far that, in schizophrenia patients, GABAergic inhibition is selectively but consistently reduced in the prefrontal cortex (PFC).

**Results:**

This study used a computational model of the PFC to investigate the dynamics of the PFC circuit with and without chandelier cells and other GABAergic interneurons. The inhibition by GABAergic interneurons other than chandelier cells effectively regulated the PFC activity with rather low or modest levels of dopaminergic neurotransmission. This activity of the PFC is associated with normal cognitive functions and has an inverted-U shaped profile of dopaminergic modulation. In contrast, the chandelier cell-type inhibition affected only the PFC circuit dynamics in hyperdopaminergic conditions. Reduction of chandelier cell-type inhibition resulted in bistable dynamics of the PFC circuit, in which the upper stable state is associated with a hyperactive mode. When both types of inhibition were reduced, this hyperactive mode and the conventional inverted-U mode merged.

**Conclusion:**

The results of our simulation suggest that, in schizophrenia, a reduction of GABAergic inhibition increases vulnerability to psychosis by (i) producing the hyperactive mode of the PFC with hyperdopaminergic neurotransmission by dysfunctional chandelier cells and (ii) increasing the probability of the transition to the hyperactive mode from the conventional inverted-U mode by dysfunctional GABAergic interneurons.

## Background

A number of studies have suggested alterations of the gamma-aminobutyric acid (GABA) system in the brains of patients with schizophrenia (for reviews: [1-5]). The alteration of GABAergic neurotransmission in the cortex seems to be selective for subpopulations of the interneurons [4-7]. Postmortem studies by Benes and colleagues reported decreased densities of interneurons in layer II of the prefrontal cortex (PFC) and layers II-IV of the cingulate cortex of patients with schizophrenia [8,9]. Possibly owing to its compensation, GABA_A _receptors were observed to be upregulated in layers II, III, V and VI in the PFC and layers II and III in the cingulate cortex [10,11]. Decreased densities of the interneurons in the PFC and the cingulate cortex might be restricted to the interneurons expressing calbindin; whether the densities of calretinin (CR)- or parvalbumin (PV)-interneurons are reduced or not is still uncertain [6,12-14].

Analyses of postmortem brains of patients with schizophrenia have shown consistent reduction of reelin, PV, and GAD67, the 67-kilodalton isoform of glutamic acid decarboxylase [15-17]. Reelin is secreted preferentially by cortical GABAergic interneurons in layers I, II and IV and binds to integrin receptors on dendritic spines of pyramidal neurons or on GABAergic interneurons in layers III-V expressing the disabled-1 gene product (DAB1) [15,18]. The expression of reelin mRNA was decreased in GABAergic interneurons in layers I, II and IV of schizophrenia patients [19]. Because reelin plays a role in neuronal migration and synaptic plasticity in the cerebral cortex [18,20,21], the reduction of reelin in schizophrenia would indicate a neurodevelopmental abnormality that induces a GABAergic deficit in schizophrenia [1,21]. Reduced levels of mRNA for GAD67 in the dorsolateral prefrontal cortex (DLPFC) of patients with schizophrenia suggest that GABA synthesis is reduced in schizophrenia [22-25]. The reduction was detected in about 25-30% of the GABAergic interneurons in the DLPFC [25,26]. Among many subtypes of GABAergic interneurons in the cortex, the PV-interneurons contain basket cells and chandelier cells, which constitute 20-25% of GABAergic interneurons in the primate DLPFC [28]. The GABAergic interneurons that show GAD67 mRNA reduction express PV [27], suggesting that the reduction is selective. Lewis and coworkers suggested that the density of the GABA membrane transporter (GAT1)-immunoreactive axon cartridges of chandelier cells was decreased by 40% in schizophrenic subjects compared to both normal controls and subjects with other psychotic disorders [29,30]. They argued that the reduction was due to a decrease in the number of axon terminals rather than the number of chandelier cells [29,30]. In contrast, the CR-positive GABAergic interneurons, which constitute about 50%, were unaffected [3].

The regulation of GABAergic neurotransmission is critical for proper information processing in the brain. For example, Goldman-Rakic and coworkers demonstrated that iontophoretic application of bicuculline methiodide, a competitive antagonist of GABA_A _receptors, into the DLPFC of monkeys performing an oculomotor delayed response task resulted in the destruction of spatial tuning of both pyramidal neurons and GABAergic interneurons [31]. This has been reproduced in computational studies, which suggests that isodirectional intracortical inhibition contributes to the stability of the cortical circuit and cross-directional inhibition contributes to the spatial tuning or selectivity of working memory to represent [32-34]. Therefore, the alteration of GABAergic neurotransmission in the cortex would cause dysregulation of the circuit dynamics, resulting in the impairment of working memory and other cognitive functions.

A recent neurophysiological study of rats [35] suggests that chandelier cells, whose spontaneous activity is fairly low, are reserved to prevent excessive firing of neurons in the circuit. Chandelier cells are characterized by their synapses on the axonal initial segment of pyramidal neurons and the reduction of GABAergic neurotransmission in cortical circuits by this type of interneurons might lead to disinhibitory overactivation of the cortex, such as epileptic activity [36]. Given that the density of the axon terminals of chandelier cells is reduced in schizophrenia, as suggested by postmortem studies [30,37], one of the consequences of the circuit abnormality in schizophrenia would be hyperexcitability of the cortex.

Early functional imaging studies reported reduced responses of the DLPFC or hypofrontality in patients with schizophrenia [38-41]. Many recent studies suggest overactivation of the DLPFC during performing working memory tasks [42-45] or both greater and less activation of subareas in the DLPFC [46,47]. The DLPFC would be basically hypodopaminergic, according to the dopamine (DA) hypothesis of schizophrenia [48]. In this situation, the GABAergic inhibition in the DLPFC is not strong. Increasing the DA release in the DLPFC increases glutamatergic neurotransmission through N-methyl-D-aspartate (NMDA) receptors by D1 receptor stimulation. Then, the activity of the DLPFC increases with the DA release in the DLPFC. Under hyperdopaminergic conditions, the GABAergic inhibition becomes so strong that it highly suppresses noisy signal neurotransmission in the DLPFC circuit [49]. The DLPFC activity thus shows an inverted-U shaped profile of the dopaminergic modulation [50,51]. The profile would be sensitive to the strength of the GABAergic inhibition because the decreasing phase of the inverted-U shaped curve critically depends on the GABAergic inhibition in the DLPFC [49-51]. Therefore, if the GABAergic inhibition in the DLPFC is weakened, as has been observed in schizophrenia, the activity of the DLPFC would be significantly different. In this case, neurons in the DLPFC would exhibit hyperexcitation due to high NMDA currents under hyperdopaminergic conditions [49,52].

Psychostimulants generally increase DA release from dopaminergic neurons [53]. Psychotic states induced by psychostimulants are accompanied by the focal activation of the PFC, and the activity has a positive correlation with a psychotic symptom [54,55]. Therefore, hyperdopaminergic neurotransmission and hyperactivity would characterize the PFC in acute psychotic states. The conventional inverted-U shape characteristic of dopaminergic modulation of the PFC activity [50], however, does not predict this. It rather predicts hypoactivity of the PFC with hyperdopaminergic neurotransmission. This unresolved issue would be an obstacle for advancing our understanding of the circuit mechanisms of schizophrenia. Recently, the circuit dynamics of the PFC under dopaminergic modulation has been studied using a computational model of the PFC circuit [34,56-59]. This model predicts how the circuit dynamics of the PFC varies with D1 receptor activation. The stability of the PFC circuit varies with the D1 receptor activation when the operating point of the circuit moves along the inverted-U shaped curve. Using this model, Tanaka and coworkers extended the range of the D1 receptor activation to extremely high levels, and showed that hyperactivation of the PFC can occur under hyperdopaminergic conditions (they termed this the 'H mode') [58]. Our study in this article uses essentially the same model and will explore the roles of GABAergic inhibition in the regulation of such dynamics of the PFC circuit. The result will show that 'chandelier cell-type inhibition' controls the H mode activity. GABAergic interneurons other than chandelier cells do not regulate this hyperactive mode effectively. Instead, these GABAergic interneurons regulate the conventional inverted-U shape mode of PFC activity. With these results, we will discuss the roles of GABAergic inhibition in the regulation and dysregulation of PFC circuit dynamics. The aim of this article is to investigate how the GABAergic abnormalities observed in the patients with schizophrenia alter the PFC circuit dynamics. Preliminary results have been published in an abstract form [59].

## Results

### Mode diagram of the PFC

**Figure 1:**
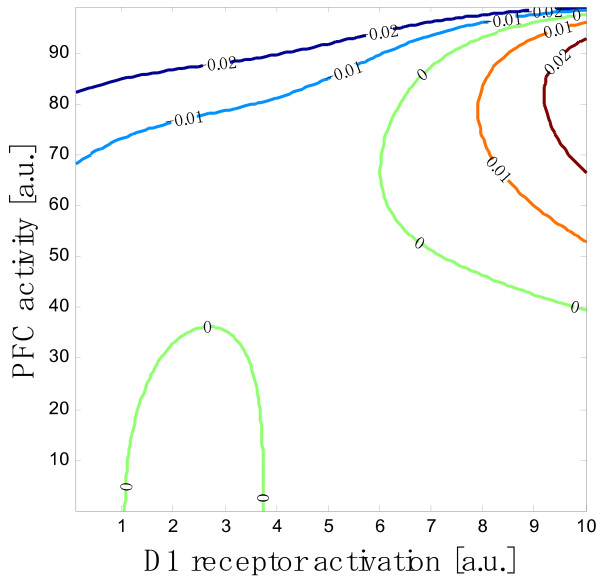
**The mode diagram of the PFC with respect to dopaminergic modulation via D1 receptors**. The vertical axis is the population activity of the pyramidal neurons in the PFC, and the horizontal axis is the D1 receptor activation level (which is denoted by *z *in the text). Only the curves numbered 0 correspond to the equilibrium state of the PFC circuit. See the text for the method of drawing of this diagram.

Equations (4) in Methods describe how the changes in the neuronal state variables (*x*_*p*_, *x*_*c *_and *x*_*n *_for pyramidal neurons, chandelier cells and other GABAergic interneurons, respectively) depend on the D1 receptor activation. To clarify the roles of the chandelier cells, we first see the circuit dynamics of the PFC without chandelier cells. Figure 1 shows the dependence of the PFC activity on the D1 receptor activation. It is a contour plot of *dx*_*p*_/*dt*, and the curves numbered 0 correspond to the equilibrium states of the pyramidal neuron population. The equilibrium state is obtained mathematically from Equations (4), by putting *dx*_*p*_/*dt *= *dx*_*c*_/*dt *= *dx*_*n*_/*dt *= 0, as

(1) *x*_*p *_= *τ*_*p *_[*W*_*pp*_(*z*) *f*_*p*_(*x*_*p*_) - *W*_*np *_*f*_*n *_{*τ*_*n*_(*z*)*W*_*pn*_(*z*) *f*_*p*_(*x*_*p*_)}]

The above equation does not contain the term for the chandelier cells (or *W*_*cp *_= 0) because we first see the circuit dynamics of the PFC without chandelier cells. The relationship between *x*_*p *_and *z *in the above equation gives the mode diagram, which is identical to the curves for the equilibrium states in Figure 1. The equilibrium state has two typical modes of the PFC activity in the different range of D1 receptor activation, *z*. One is the inverted-U mode (1.0 <*z *< 4.3) and the other is the H mode (*z *> 6). There is a gap between these modes (4.3 <*z *< 6), in which the activity of the PFC is suppressed. Beyond *z *= 6, the PFC has two branches of activity. The upper branch is stable while the lower branch is unstable, as shown below, meaning that the dynamics of the PFC circuit is bistable. Therefore, once the PFC activity becomes higher than the unstable branch, the activity increases to reach the upper stable branch, whereas PFC activity that is lower than the unstable branch decreases to zero.

### Analysis

**Figure 2:**
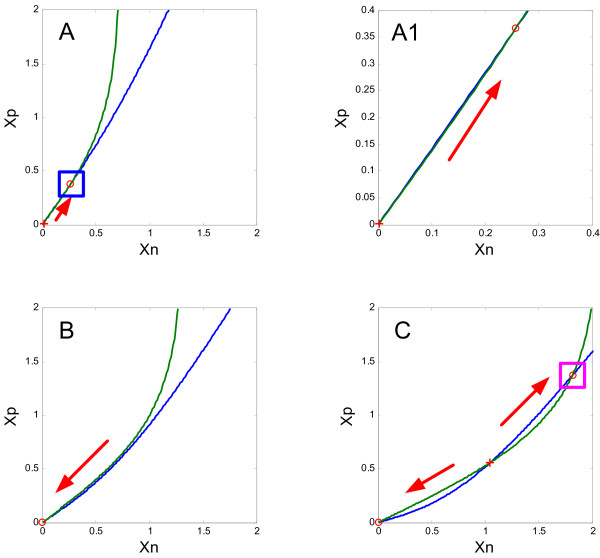
**Nullclines of the state variables of the pyramidal neurons and the GABAergic interneurons other than chandelier cells for three different levels of D1 receptor activation**. A: *z *= 3.0; B: *z *= 5.0; C: *z *= 7.0. The nullcline for the pyramidal neurons (*dx*_*p*_/*dt *= 0) is depicted in blue and the nullcline for the GABAergic interneurons (*dx*_*n*_/*dt *= 0) is depicted in green. The inset (A1) is an enlargement view of A. The circles indicate stable fixed points and the crosses indicate unstable fixed points. The arrows show the direction of state transition toward one of the stable fixed points.

One can see state transitions toward the stable equilibrium states by depicting nullclines and fixed points at typical D1 receptor activation levels. Figure 2 shows the nullclines for the state variables of the pyramidal neurons and the GABAergic interneurons other than chandelier cells, *x*_*p *_and *x*_*n*_. These nullclines are obtained by setting *dx*_*p*_/*dt *= *dx*_*n*_/*dt *= 0 as

(2) $$\begin{array}{l} x_{p}=\tau_{p}\left[W_{pp}(z)f_{p}(x_{p})-W_{np}f_{n}(x_{n})\right]\\ x_{n}=\tau_{n}(z)W_{pn}(z)f_{p}(x_{p}) \end{array}$$

These are the equilibrium conditions for the two populations of neurons. The intersections of these nullclines indicate, therefore, the equilibrium states of the whole circuit or the fixed points. The figure shows three different conditions, mentioned above; i.e., the inverted-U mode (Figure 2A), the inactive state (Figure 2B), and the H mode (Figure 2C). The inverted-U mode has a single stable fixed point, indicated by a circle in Figures 2A and 2A1. The inactive state has no intersection between the two nullclines, so that only the state *x*_*p *_= *x*_*n *_= 0 is stable. In the H mode condition, there are two intersections of the nullclines or fixed points. Among these, the lower fixed point, indicated by a cross, is unstable, whereas the higher fixed point, indicated by a circle, is stable. This stable fixed point characterizes the H mode by hyperactivity of the PFC neurons.

### Roles of GABAergic inhibition

We next investigate the roles of chandelier cells and other GABAergic interneurons. We see how the changes in the strength of GABAergic inhibition alter the PFC activity. The equilibrium condition of the PFC in this case is given by

(3) *x*_*p *_= *τ*_*p*_[*W*_*pp*_(*z*)*f*_*p*_(*x*_*p*_) - *W*_*cp*_*f*_*c *_{*τ*_*c*_(*z*) *W*_*pc*_(*z*) *f*_*p*_(*x*_*p*_)} - *W*_*np*_*f*_*n *_{*τ*_*n*_(*z*) *W*_*pn*_(*z*) *f*_*p*_(*x*_*p*_)}]

**Figure 3:**
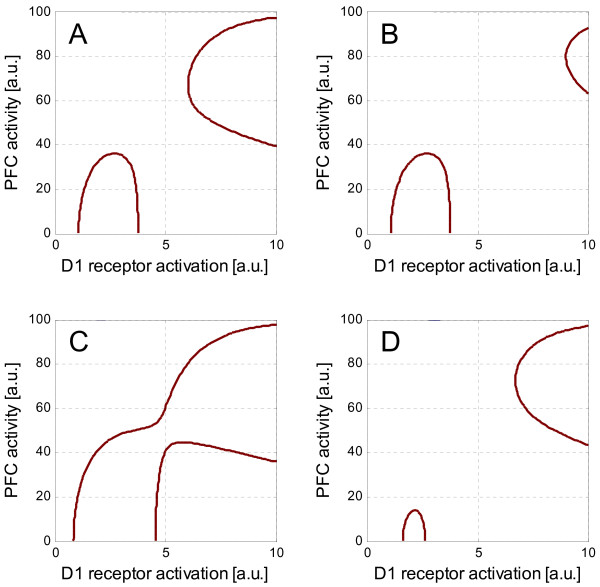
**Mode diagrams**. A: Control. B: With chandelier cells. C: Weaker GABAergic interneurons other than chandelier cells. D: Stronger GABAergic interneurons other than chandelier cells. The chandelier cells are dysfunctional in A, C and D.

**Figure 4:**
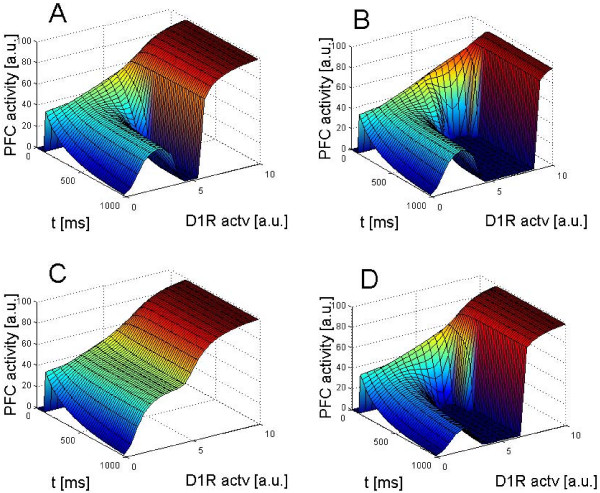
**Three-dimensional representations of DA modulatory landscapes with (B) and without (A, C, and D) chandelier cells**. The strength of the inhibition by other GABAergic interneurons are also varied (A: 1.0, B: 1.0, C: 0.95, and D: 1.06). Note that the onset of the H mode is very quick (less than 100 ms), whereas the inverted-U mode profiles are very slow to evolve. Even at *t *= 1000 ms, the profiles of the inverted-U mode have not reached the equilibrium states. The profiles at equilibrium are shown in Figure 3.

The results are depicted in Figure 3, which are mode diagrams for different levels of GABAergic inhibition. Figure 3A is the same with Figure 1. In Figure 3B, the inhibition by the chandelier cells is increased, which moves the H mode away from the inverted-U mode without altering the inverted-U mode profile. When the inhibition by the GABAergic interneurons other than chandelier cells becomes weaker and the chandelier cells are dysfunctional, the inverted-U mode and the H mode are connected (Figure 3C). Stronger inhibition, on the other hand, shrinks the inverted-U mode but does not affect the H mode significantly (Figure 3D). This means that the inverted-U mode, but not the H mode, is sensitive to this type of inhibition. A further increase in this type of inhibition eliminates the inverted-U mode. In contrast, the H mode is robust against this type of inhibition; only the chandelier cells can separate it from the inverted-U mode. Figure 4 shows the three-dimensional views of the temporal evolutions of these profiles. The variations of the parameter values used in the simulation are summarized in Table 1.

**Table 1:** A summary of the variations of the parameter values of the two different types of GABAergic inhibition used in the simulation.

	Chandelier cells	Other GABA neurons
	
A	decreased (0.0)	unchanged (1.0)
B	control (1.0)	control (1.0)
C	decreased (0.0)	decreased (0.95)
D	decreased (0.0)	increased (1.06)

The figures in the parentheses indicate the relative strength of the inhibitory action on the pyramidal neurons in the model PFC circuit.

## Discussion

### Chandelier cells vs other GABAergic interneurons

Our computational studies suggest that the dopaminergic modulation profile of PFC activity is complex rather than just an inverted U. A remarkable thing is the possibility of the existence of the H mode or hyperactive mode of the PFC with hyperdopaminergic neurotransmission. Both this mode and the conventional inverted-U mode activity of the PFC under the dopaminergic modulation would be regulated by GABAergic neurotransmission. However, the simulation in this article suggests that these modes have different sensitivities to different types of GABAergic inhibition. The H mode is sensitive to the GABAergic inhibition by chandelier cells, whereas the inverted-U mode is sensitive to the inhibition by GABAergic interneurons other than chandelier cells. The emergence of the H mode is, therefore, critically dependent on the strength of the chandelier cell-type inhibition. Stronger inhibition of this type puts the H mode away from the inverted-U mode. This means that, when the GABAergic inhibition by chandelier cells is reduced, as suggested in schizophrenia, the H mode is considered to be closer to the inverted-U mode than in healthy controls. On the other hand, the profile of the inverted-U mode is critically dependent on the inhibition by GABAergic interneurons other than chandelier cells. If this type of inhibition is stronger, the inverted-U mode easily disappears. With weaker inhibition of this type, in contrast, the profile of the inverted-U mode becomes larger. If both types of inhibition are reduced, therefore, the inverted-U mode and the H mode would merge into a single mode. As a result, the state of the PFC would be able to move to the H mode from the inverted-U mode.

### Transition to the H mode

**Figure 5:**
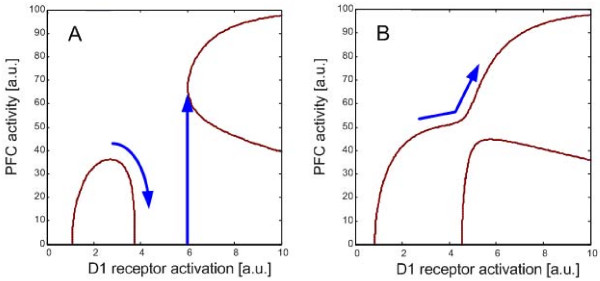
**Mode diagrams and state transition**. A: When the chandelier cells are dysfunctional but the GABAergic inhibition by the other interneurons is normal, the transition from the inverted-U mode to the H mode hardly occurs because of a gap between the two modes. The gap becomes wider in the existence of chandelier cells. B: When the chandelier cells are dysfunctional and the GABAergic inhibition by the other interneurons is reduced, the two modes are connected, so that the transition to the H mode would occur readily.

The transition from the inverted-U mode to the H mode is illustrated by Figure 5. When the two modes are separated (Figure 5A), the inverted-U mode activity decreases as D1 receptors are activated further. Then, it would be difficult to cross the gap to reach the H mode. Once they are connected (Figure 5B), however, it would be much easier to reach the H mode from the inverted-U mode by, for example, increasing the D1 receptor activation. The transition from the inverted-U mode to the H mode would have important relevance to schizophrenia. First, the H mode would be associated with psychotic states, as will be argued below. Second, chandelier cells would prevent the occurrence of psychotic states by suppressing the H mode activity. Third, weakening of the inhibition by other GABAergic interneurons increases the probability of the transition to the H mode or vulnerability to psychosis. These would explain the reason why schizophrenic brains are vulnerable to psychosis and are consistent with the finding of the reduced GABAergic inhibition in the PFC of patients with schizophrenia.

### Psychosis

#### Schizophrenia

Functional magnetic resonance imaging (fMRI) studies of patients with schizophrenia using a verbal fluency task showed that increasing task demand produced greater activation of the PFC with higher error rates in psychotic states compared with remission [60]. A recent fMRI study suggested an association between reality distortion and hyperactivity of the medial PFC of patients with schizophrenia or schizoaffective disorders [61]. Besides these, 'it is postulated that before experiencing psychosis, patients [with schizophrenia] develop an exaggerated release of DA, independent of and out of synchrony with the context' [62]. Downregulation of GABAergic neurotransmission in the PFC has consistently been associated with schizophrenia [1-5,15]. These support our theory that psychotic states are induced by the transition to the H mode due to reduced GABAergic inhibition in the PFC with hyperdopaminergic neurotransmission.

#### Substance-induced psychosis

Ketamine and amphetamines induced focal activation of the PFC in healthy subjects [54,63,64] and in patients with schizophrenia [65]. In either schizophrenia or drug addiction, therefore, psychosis is associated with selective or focal activation of the cortex [54,63-65]. NMDA antagonists, such as phencyclidine and ketamine, increase the extracellular DA concentration in the PFC [66-68]. It has been suggested that acute administration of psychostimulants, such as amphetamines and cocaine, increases the extracellular DA level significantly not only in the subcortical areas but in the PFC [69,70]. A microdialysis study reported that intraperitoneal administration of 2 mg/kg of amphetamine to rats induced six-fold increase in the baseline DA concentration in the PFC [69], which could activate D1 receptors in the PFC. Recent studies reported that ketamine, an NMDA antagonist, decreased the expression of PV and GAD67 in mice [71,72], suggesting reduced GABAergic inhibition in the PFC. Therefore, the underlying circuit mechanism of substance-induced psychosis might be the same with schizophrenic psychosis; that is, the transition to the H mode due to reduced GABAergic inhibition in the PFC with hyperdopaminergic neurotransmission.

### Dopamine-mediated mechanisms

#### Upregulation of D1 receptors

In contrast to acute administration, chronic administration of psychostimulants lowers the extracellular concentration of DA in the PFC [73,74], which would induce sensitization of DA receptors. Similarly, the sensitization of DA receptors would be expected in patients with schizophrenia. A positron emission tomography (PET) study, using [11C]NNC 112 as a radiotracer, observed an increase in the binding potential of D1 receptors in the PFC of schizophrenia patients [75]. This would reflect a chronically reduced extracellular DA concentration and an increase in the density of D1 receptors. Upregulation or sensitization of D1 receptors might be involved in schizophrenia. An increase in the DA releasability or the responsivity of dopaminergic neurons has also been suggested [76,77]. These situations would increase the *z *value in the model, thereby increasing susceptibility to the H mode.

#### Stress

Acute stress increases DA turnover in the PFC, which leads to the impairment of cognitive functions [78,79]. It seems that metabolic activity of dopaminergic neurons innervating the PFC is increased selectively in the PFC [80]. The administration of the stressor FG 7142 also increases DA turnover in the PFC [81,82]. Chronic stress induced hypodopaminergic states, and, again, impaired cognitive functions [83]. In this case, *B*_max _or the density of D1 receptors in rat PFC was significantly increased (from 14.5 with 2.9 SD to 22.3 with 3.5 SD). Interestingly, either the hyperdopaminergic state or the hypodopaminergic state with D1 upregulation could lead to the H mode, according to the above arguments.

#### Epilepsy

People with epilepsy are susceptible to schizophrenia-like psychosis [84-86]. The association between epilepsy and schizophrenia-like psychosis has long attracted much attention [87,88], and would be interesting to know the commonalities between epilepsy and schizophrenia and the mechanisms underlying both diseases. Epilepsy is accompanied by excessive excitation of neuronal circuits in the brain [89,90]. Many studies have suggested selective alterations in GABA_A _receptor subtypes in patients with epilepsy [91,92]. DeFelipe proposed the hypothesis that the chandelier cell is a key component of cortical circuits in the establishment of epilepsy [36]. Links to dopaminergic mechanisms have also been suggested [93,94]. Using whole-cell recording and voltage-sensitive dye imaging techniques in the rat PFC, Bandyopadhyay et al. [95] demonstrated that bath application of SKF 81297, a selective D1 receptor agonist, enhanced spatiotemporal spread of activity in response to weak stimulation and previously subthreshold stimulation resulted in epileptiform activity that spread across the whole cortex. This result indicates that DA, via a D1 receptor-mediated mechanism, enhances spatiotemporal spread of neuronal activity and lowers the threshold for epileptiform activity in local circuits within the PFC. A rat study suggested that the supersensitivity of the DA systems, which was developed in the chronic phase of the kainate-induced temporal lobe epilepsy, was responsible for the genesis of epileptic psychosis [93]. The H mode hypothesis is consistent with all of these results.

#### Enhanced cortical inputs

Because of the bistable nature of the H mode, the occurrence of the H mode critically depends on the strength of inputs. They are mediated by corticocortical or thalamocortical afferents to the PFC, and would be modulated by several ways, including dopaminergic modulation. It has also been suggested that DA has a sensorimotor gating function in PFC and subcortical circuits [96-99]. In fact, many studies have reported deficits in the sensorimotor gating function in patients with schizophrenia (for reviews: [98,100,101]) and, interestingly, also in amphetamine-sensitized animals [102]. When a dysregulated or unfiltered input is given to the PFC, the PFC would respond to it with hyperactivity. Recent neurophysiological study in monkey reported an enhancement of the response-period activity of DLPFC neurons, but no effect on delay-period activity, by the stimulation of the D2 receptors in the DLPFC [103]. This may suggest that D2 receptors are involved in gating afferent input to the DLPFC circuit for working memory and other cognitive functions. Moreover, if D2 receptors are supersensitive [104,105], the H mode would more readily emerge because hyperactivation of D2 receptors could contribute to the enhancement of the input to the PFC.

## Conclusion

We have investigated how GABAergic inhibition by chandelier cells and other GABAergic interneurons contribute to the regulation of neuronal activity in the PFC circuit. The results show that the roles of the two different types of GABAergic inhibition on PFC circuit dynamics are markedly different. The inhibition by GABAergic interneurons other than chandelier cells effectively regulates the PFC activity with rather low or modest levels of dopaminergic neurotransmission, which has an inverted-U shaped profile of dopaminergic modulation and is associated with normal cognitive functions. In contrast, the chandelier cell-type inhibition regulates the PFC activity with hyperdopaminergic neurotransmission. Therefore, dysfunction of chandelier cells in the PFC would produce the H mode, a "psychotic" hyperactive state with hyperdopaminergic neurotransmission. Reduction of the inhibition by other GABAergic interneurons would make the transition to the H mode more readily occur, thereby increasing vulnerability to psychosis.

## Methods

### Prefrontal Cortical Circuit Model

**Figure 6:**
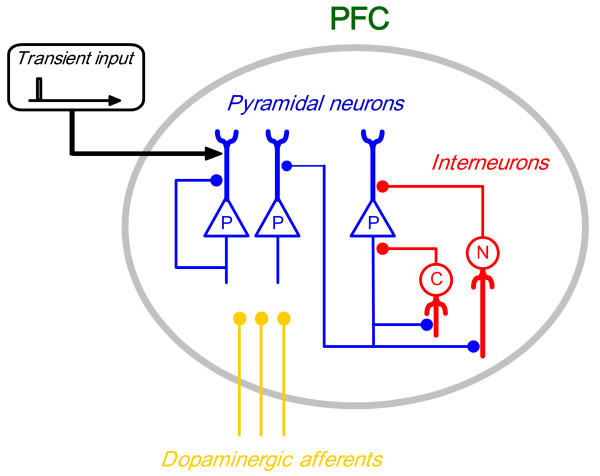
**A schematic diagram of the model**. The PFC contains pyramidal neurons and GABAergic interneurons (chandelier cells (C) and others (N)), which are connected reciprocally and have also self-innervations. All populations of neurons are under dopaminergic modulation via D1 receptors. The transient input to the pyramidal neurons triggers the dynamics of the circuit.

Our model of the PFC contains pyramidal neurons and GABAergic interneurons (Figure 6). The pyramidal neurons have recurrent connections or self-innervations. The two populations of neurons are connected reciprocally. All of the neurons in the model are assumed to be under dopaminergic modulation via D1 receptors; D1 receptor activation changes the synaptic strengths from pyramidal neurons to both pyramidal neurons and interneurons as well as the time constant for the interneurons [34,106]. The dopaminergic modulation via D1 receptors in this model is consistent with that of Durstewitz et al. [107] but is a reduced one that is suitable for the present analysis with the firing rate model.

The pyramidal neurons receive a transient external input, which triggers the dynamics of the circuit. Our model describes the activity of each population of neurons (either pyramidal neurons or GABAergic interneurons) by a single state variable, which therefore describes the population activity. The state equations for the population activities are given by

(4) $$\begin{array}{l} \frac{dx_{p}}{dt}=-\frac{x_{p}}{\tau_{p}}+W_{pp}(z)f_{p}(x_{p})-W_{cp}f_{c}(x_{c})-W_{np}f_{n}(x_{n})+I_{cue}\\ \frac{dx_{c}}{dt}=-\frac{x_{c}}{\tau_{c}(z)}+W_{pc}(z)f_{p}(x_{p})\\ \frac{dx_{n}}{dt}=-\frac{x_{n}}{\tau_{n}(z)}+W_{pn}(z)f_{p}(x_{p}) \end{array}$$

where *x*_*p*_, *x*_*c *_and *x*_*n *_are the state variables for the pyramidal neuron population, the chandelier cell population, and the population of the GABAergic interneurons other than chandelier cells, *p*, *c *and *n *denote the pyramidal neurons, the chandelier cells, and the GABAergic interneurons other than chandelier cells, respectively, *τ*_*p*_, *τ*_*c *_and *τ*_*n *_are the time constants of these neurons, *W*_*ij *_(*i*, *j *= *p*, *c*, *n*) is the synaptic efficacy from population *i *to *j*, and *I*_*cue *_is the transient external input to the pyramidal neuron population. The parameters that depend on *z *are subject to dopaminergic modulation, where *z *is the D1 receptor activation (see below). The activation function is assumed to be common to the populations of pyramidal neurons and GABAergic interneurons other than chandelier cells:

(5) $$f_{p}(x)=f_{n}(x)=\{\begin{array}{cc} f_{\max}\tanh(x) & x\geq 0\\ 0 & x<0 \end{array}$$

where *f*_max _is the maximum firing rate. The activation function for the chandelier cell population will be given below. The simulation used the values of the parameters in the above equations as: *f*_max _= 100 sp/s, *τ*_*p *_= 20.0, *τ*_*c *_(0) = *τ*_*n *_(0) = 5.0, *W*_*pp *_(0) = 0.00055, *W*_*pc *_(0) = *W*_*pn *_(0) = 0.00035, *W*_*cp *_= 0.0002, and *W*_*np *_= 0.0005.

### GABAergic Interneurons

Spontaneous activity of chandelier cells is fairly low but they fire action potentials at frequencies higher than other GABAergic interneurons when the overall cortical excitation increases, suggesting that their role is to suppress excessive excitation via their powerful inhibitory synapses on pyramidal neurons [35,108]. With their unique synapses on the axonal initial segment, chandelier cells would increase their inhibitory effects when the GABA release from chandelier cell axon terminals becomes coincident with spike generation of the postsynaptic pyramidal neurons. This would require highly repetitive inputs from pyramidal neurons so that chandelier cells can fire at a high rate. Therefore, the inhibitory effect by the chandelier cell would increase sharply when the firing rate exceeds a certain threshold. We describe this characteristic of inhibitory effect by chandelier cells simply with an activation function

**Figure 7:**
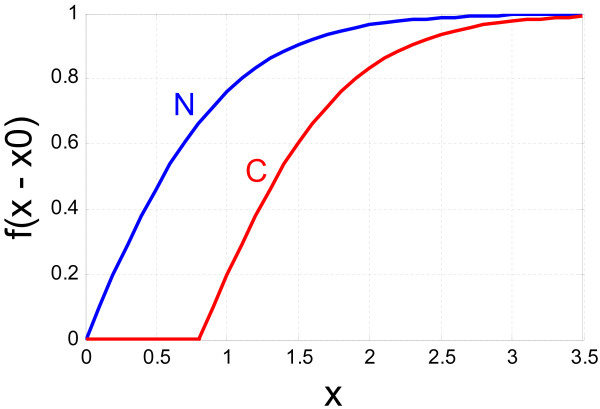
The profiles of the activation functions of the chandelier cells (C) and the other GABAergic interneurons (N) in the model.

(6) *f*_*c *_(*x*) = *f*_max _tanh(*x *- *x*_0_)

where *x*_0 _= 0.8 is the threshold above which the inhibition by the chandelier cell becomes effective. Figure 7 shows the profiles of the activation functions for the populations of the chandelier cells and other GABAergic interneurons. The difference in physiological properties between these populations of neurons exists only in the thresholds in the activation functions. For a network model consisting of different types of interneurons with Hodgkin-Huxley models, refer to [109], which studied differential contributions to working memory representation in the DLPFC. It would be interesting to see how the subtypes of interneurons affect the profile of PFC activity under dopaminergic modulation.

### Dopaminergic Modulation via D1 Receptors

The activation of D1 receptors affects the channel conductances, such as the conductances of α-amino-3-hydroxy-5-methyl-4-isoxazole propionic acid (AMPA) and NMDA receptor-channels (for reviews: [110,111]). These change the efficacy of glutamatergic signal neurotransmission (*W*_*pp*_, *W*_*pc *_and *W*_*pn *_in this model). The excitability of the PFC inhibitory interneurons increases with D1 receptor activation by decreasing the potassium-channel conductance [112]. This leads to a model in which the time constants of the interneurons, *τ*_*c *_and *τ*_*n*_, are assumed to decrease with D1 receptor activation [34,106]. Taken together, our model describes these effects by

(7) $$\begin{array}{l} W_{pp}(z)=W_{pp}(0)(1+az)\\ W_{pc}(z)=W_{pc}(0)(1+bz)\\ W_{pn}(z)=W_{pn}(0)(1+bz)\\ \tau_{c}(z)=\tau_{c}(0)(1+cz)\\ \tau_{n}(z)=\tau_{n}(0)(1+cz) \end{array}$$

where *a*, *b *and *c *are constants (*a *= 0.2, *b *= 0.4, *c *= 0.3).

## Authors' contributions

ST carried out the design of the study, modeling, computer simulation, the analysis of the results, and manuscript preparation.

## References

[B1] Benes FM, Berretta S (2001). GABAergic interneurons: implications for understanding schizophrenia and bipolar disorder. Neuropsychopharmacology.

[B2] Lewis DA, Gonzalez-Burgos G (2006). Pathophysiologically based treatment interventions in schizophrenia. Nat Med.

[B3] Lewis DA, Moghaddam B (2006). Cognitive dysfunction in schizophrenia: convergence of gamma-aminobutyric acid and glutamate alterations. Arch Neurol.

[B4] Reynolds GP, Zhang ZJ, Beasley CL (2001). Neurochemical correlates of cortical GABAergic deficits in schizophrenia: selective losses of calcium binding protein immunoreactivity. Brain Res Bull.

[B5] Guidotti A, Auta J, Davis JM, Dong E, Grayson DR, Veldic M, Zhang X, Costa E (2005). GABAergic dysfunction in schizophrenia: new treatment strategies on the horizon. Psychopharmacology (Berl).

[B6] Beasley CL, Zhang ZJ, Patten I, Reynolds GP (2002). Selective deficits in prefrontal cortical GABAergic neurons in schizophrenia defined by the presence of calcium-binding proteins. Biol Psychiatry.

[B7] Reynolds GP, Beasley CL (2001). GABAergic neuronal subtypes in the human frontal cortex – development and deficits in schizophrenia. J Chem Neuroanat.

[B8] Benes FM (2000). Emerging principles of altered neural circuitry in schizophrenia. Brain Res Rev.

[B9] Benes FM, McSparren J, Bird ED, SanGiovanni JP, Vincent SL (1991). Deficits in small interneurons in prefrontal and cingulate cortices of schizophrenic and schizoaffective patients. Arch Gen Psychiatry.

[B10] Benes FM, Vincent SL, Alsterberg G, Bird ED, SanGiovanni JP (1992). Increased GABAA receptor binding in superficial layers of cingulate cortex in schizophrenics. J Neurosci.

[B11] Benes FM, Vincent SL, Marie A, Khan Y (1996). Up-regulation of GABAA receptor binding on neurons of the prefrontal cortex in schizophrenic subjects. Neuroscience.

[B12] Cotter D, Landau S, Beasley C, Stevenson R, Chana G, MacMillan L, Everall I (2002). The density and spatial distribution of gabaergic neurons, labelled using calcium binding proteins, in the anterior cingulate cortex in major depressive disorder, bipolar disorder, and schizophrenia. Biological Psychiatry.

[B13] Reynolds GP, Beasley CL, Zhang ZJ (2002). Understanding the neurotransmitter pathology of schizophrenia: selective deficits of subtypes of cortical GABAergic neurons. J Neural Transm.

[B14] Woo TU, Miller JL, Lewis DA (1997). Schizophrenia and the parvalbumin-containing class of cortical local circuit neurons. Am J Psychiatry.

[B15] Guidotti A, Auta J, Davis JM, Di-Giorgi-Gerevini V, Dwivedi Y, Grayson DR, Impagnatiello F, Pandey G, Pesold C, Sharma R, Uzunov D, Costa E (2000). Decrease in reelin and glutamic acid decarboxylase67 (GAD67) expression in schizophrenia and bipolar disorder: a postmortem brain study. Arch Gen Psychiatry.

[B16] Impagnatiello F, Guidotti AR, Pesold C, Dwivedi Y, Caruncho H, Pisu MG, Uzunov DP, Smalheiser NR, Davis JM, Pandey GN, Pappas GD, Tueting P, Sharma RP, Costa E (1998). A decrease of reelin expression as a putative vulnerability factor in schizophrenia. Proc Natl Acad Sci USA.

[B17] Torrey EF, Barci BM, Webster MJ, Bartko JJ, Meador-Woodruff JH, Knable MB (2005). Neurochemical markers for schizophrenia, bipolar disorder, and major depression in postmortem brains. Biol Psychiatry.

[B18] Pesold C, Impagnatiello F, Pisu MG, Uzunov DP, Costa E, Guidotti A, Caruncho HJ (1998). Reelin is preferentially expressed in neurons synthesizing gamma-aminobutyric acid in cortex and hippocampus of adult rats. Proc Natl Acad Sci USA.

[B19] Veldic M, Caruncho HJ, Liu WS, Davis J, Satta R, Grayson DR, Guidotti A, Costa E (2004). DNA-methyltransferase 1 mRNA is selectively overexpressed in telencephalic GABAergic interneurons of schizophrenia brains. Proc Natl Acad Sci USA.

[B20] Herz J, Chen Y (2006). Reelin, lipoprotein receptors and synaptic plasticity. Nat Rev Neurosci.

[B21] Tueting P, Doueiri MS, Guidotti A, Davis JM, Costa E (2006). Reelin down-regulation in mice and psychosis endophenotypes. Neurosci Biobehav Rev.

[B22] Akbarian S, Huang HS (2006). Molecular and cellular mechanisms of altered GAD1/GAD67 expression in schizophrenia and related disorders. Brain Res Rev.

[B23] Lewis DA, Hashimoto T, Volk DW (2005). Cortical inhibitory neurons and schizophrenia. Nat Rev Neurosci.

[B24] Volk DW, Lewis DA (2002). Impaired prefrontal inhibition in schizophrenia: relevance for cognitive dysfunction. Physiol Behav.

[B25] Volk DW, Austin MC, Pierri JN, Sampson AR, Lewis DA (2000). Decreased glutamic acid decarboxylase67 messenger RNA expression in a subset of prefrontal cortical gamma-aminobutyric acid neurons in subjects with schizophrenia. Arch Gen Psychiatry.

[B26] Akbarian S, Kim JJ, Potkin SG, Hagman JO, Tafazzoli A, Bunney WE, Jones EG (1995). Gene expression for glutamic acid decarboxylase is reduced without loss of neurons in prefrontal cortex of schizophrenics. Arch Gen Psychiatry.

[B27] Hashimoto T, Volk DW, Eggan SM, Mirnics K, Pierri JN, Sun Z, Sampson AR, Lewis DA (2003). Gene expression deficits in a subclass of GABA neurons in the prefrontal cortex of subjects with schizophrenia. J Neurosci.

[B28] Zaitsev AV, Gonzalez-Burgos G, Povysheva NV, Kroner S, Lewis DA, Krimer LS (2004). Localization of calcium-binding proteins in physiologically and morphologically characterized interneurons of monkey dorsolateral prefrontal cortex. Cereb Cortex.

[B29] Lewis DA (2000). GABAergic local circuit neurons and prefrontal cortical dysfunction in schizophrenia. Brain Res Rev.

[B30] Woo T-U, Whitehead RE, Melchitzky DS, Lewis DA (1998). A subclass of prefrontal gamma-aminobutyric acid axon terminals are selectively altered in schizophrenia. Proc Natl Acad Sci USA.

[B31] Rao SG, Williams GV, Goldman-Rakic PS (2000). Destruction and creation of spatial tuning by disinhibition: GABA(A) blockade of prefrontal cortical neurons engaged by working memory. J Neurosci.

[B32] Tanaka S (1999). Architecture and dynamics of the primate prefrontal cortical circuit for spatial working memory. Neural Networks.

[B33] Tanaka S (2000). Roles of intracortical inhibition in the formation of spatially tuned delay-period activity of prefrontal neurons: computational study. Prog Neuro-Psychopharm & Biol Psychiat.

[B34] Tanaka S (2006). Dopaminergic control of working memory and its relevance to schizophrenia: a circuit dynamics perspective. Neuroscience.

[B35] Zhu Y, Stornetta RL, Zhu JJ (2004). Chandelier cells control excessive cortical excitation: characteristics of whisker-evoked synaptic responses of layer 2/3 nonpyramidal and pyramidal neurons. J Neurosci.

[B36] DeFelipe J (1999). Chandelier cells and epilepsy. Brain.

[B37] Pierri JN, Chaudry AS, Woo T-UW, Lewis DA (1999). Alterations in chandelier neuron axon terminals in the prefrontal cortex of schizophrenic subjects. Am J Psychiatry.

[B38] Andreasen NC, Rezai K, Alliger R, Swayze VW, Flaum M, Kirchner P, Cohen G, O'Leary DS (1992). Hypofrontality in neuroleptic-naive patients and in patients with chronic schizophrenia. Assessment with xenon 133 single-photon emission computed tomography and the Tower of London. Arch Gen Psychiatry.

[B39] Carter CS, Perlstein P, Ganguli R, Brar J, Mintun M, Cohen JD (1998). Functional hypofrontality and working memory dysfunction in schizophrenia. Am J Psychiatry.

[B40] Paulman RG, Devous MD, Gregory RR, Herman JH, Jennings L, Bonte FJ, Nasrallah HA, Raese JD (1990). Hypofrontality and cognitive impairment in schizophrenia: dynamic single-photon tomography and neuropsychological assessment of schizophrenic brain function. Biol Psychiatry.

[B41] Weinberger DR, Berman KF (1988). Speculation on the meaning of cerebral metabolic hypofrontality in schizophrenia. Schizophr Bull.

[B42] Manoach DS (2003). Prefrontal cortex dysfunction during working memory performance in schizophrenia: reconciling discrepant findings. Schizophr Res.

[B43] Manoach DS, Press DZ, Thangaraj V, Searl MM, Goff DC, Halpern E, Saper CB, Warach S (1999). Schizophrenic subjects activate dorsolateral prefrontal cortex during a working memory task, as measured by fMRI. Biological Psychiatry.

[B44] Manoach DS, Gollub RL, Benson ES, Searl MM, Goff DC, Halpern E, Saper CB, Rauch SL (2000). Schizophrenic subjects show aberrant fMRI activation of dorsolateral prefrontal cortex and basal ganglia during working memory performance. Biol Psychiatry.

[B45] Thermenos HW, Goldstein JM, Buka SL, Poldrack RA, Koch JK, Tsuang MT, Seidman LJ (2005). The effect of working memory performance on functional MRI in schizophrenia. Schizophr Res.

[B46] Callicott JH, Bertolino A, Mattay VS, Langheim FJ, Duyn J, Coppola R, Goldberg TE, Weinberger DR (2000). Physiological dysfunction of the dorsolateral prefrontal cortex in schizophrenia revisited. Cereb Cortex.

[B47] Callicott JH, Mattay VS, Verchinski BA, Marenco S, Egan MF, Weinberger DR (2003). Complexity of prefrontal cortical dysfunction in schizophrenia: more than up or down. Am J Psychiatry.

[B48] Kahn RS, Davis KL (2000). New developments in dopamine and schizophrenia. Psychopharmacology – The Fourth Generation of Progress.

[B49] Williams GV, Castner SA (2006). Under the curve: critical issues for elucidating D1 receptor function in working memory. Neuroscience.

[B50] Goldman-Rakic PS, Muly EC, Williams GV (2000). D1 receptors in prefrontal cells and circuits. Brain Res Rev.

[B51] Muly EC, Szigeti K, Goldman-Rakic PS (1998). D1 receptor in interneurons of macaque prefrontal cortex: distribution and subcellular localization. J Neurosci.

[B52] Seamans JK, Durstewitz D, Christie B, Stevens CF, Sejnowski TJ (2001). Dopamine D1/D5 receptor modulation of excitatory synaptic inputs to layer V prefrontal cortical neurons. Proc Natl Acad Sci USA.

[B53] Feldman RS, Meyer JS, Quenzer LF (1997). Principles of neuropsychopharmacology.

[B54] Breier A, Malhotra AK, Pinals DA, Weisenfeld NI, Pickar D (1997). Association of ketamine-induced psychosis with focal activation of the prefrontal cortex in healthy volunteers. Am J Psychiatry.

[B55] Vollenweider FX, Leenders KL, Scharfetter C, Antonini A, Maguire P, Missimer J, Angst J (1997). Metabolic hyperfrontality and psychopathology in the ketamine model of psychosis using positron emission tomography (PET) and [18F]fluorodeoxyglucose (FDG). Eur Neuropsychopharmacol.

[B56] Tanaka S, Kuei-Yuan Tseng, Marco Atzori (2007). Stable and unstable activation of the prefrontal cortex with dopaminergic modulation. Monoaminergic Modulation of Cortical Excitability.

[B57] Tanaka S, Akiyama Watanabe (2007). A circuit dynamics theory of complex dopaminergic modulation of prefrontal cortical activity and its relevance to schizophrenia. Dopamine Research Advances.

[B58] Tanaka S, Ebi H, Yamashita K (2006). A new mode beyond the inverted-U region of the dopaminergic modulation of the prefrontal cortex. Neurocomputing.

[B59] Tanaka S, Sakata K, Horiguchi R, Ebi H (2007). A specific role of chandelier cell-type inhibition in prefrontal cortical circuit dynamics: implications for schizophrenia. Soc Neurosci Abstr.

[B60] Fu CH, Suckling J, Williams SC, Andrew CM, Vythelingum GN, McGuire PK (2005). Effects of psychotic state and task demand on prefrontal function in schizophrenia: an fMRI study of overt verbal fluency. Am J Psychiatry.

[B61] Taylor SF, Welsh RC, Chen AC, Velander AJ, Liberzon I (2007). Medial frontal hyperactivity in reality distortion. Biol Psychiatry.

[B62] Kapur S (2003). Psychosis as a state of aberrant salience: a framework linking biology, phenomenology, and pharmacology in schizophrenia. Am J Psychiatry.

[B63] Mattay VS, Berman KF, Ostrem JL, Esposito G, Van Horn JD, Bigelow LB, Weinberger DR (1996). Dextroamphetamine enhances "neural network-specific" physiological signals: a positron-emission tomography rCBF study. J Neurosci.

[B64] Uftring SJ, Wachtel SR, Chu D, McCandless C, Levin DN, Wit Hd (2001). An fMRI study of the effect of amphetamine on brain activity. Neuropsychopharmacology (Nature).

[B65] Daniel DG, Weinberger DR, Jones DW, Zigun JR, Coppola R, Handel S, Bigelow LB, Goldberg TE, Berman KF, Kleinman JE (1991). The effect of amphetamine on regional cerebral blood flow during cognitive activation in schizophrenia. J Neurosci.

[B66] Adams B, Moghaddam B (1998). Corticolimbic dopamine neurotransmission is temporally dissociated from the cognitive and locomotor effects of phencyclidine. J Neurosci.

[B67] Hondo H, Yonezawa Y, Nakahara T, Nakamura K, Hirano M, Uchimura H, Tashiro N (1994). Effect of phencyclidine on dopamine release in the rat prefrontal cortex; an in vivo microdialysis study. Brain Res.

[B68] Verma A, Moghaddam B (1996). NMDA receptor antagonists impair prefrontal cortex function as assessed via spatial delayed alternation performance in rats: modulation by dopamine. J Neurosci.

[B69] Shoblock JR, Maisonneuve IM, Glick SD (2004). Differential interactions of desipramine with amphetamine and methamphetamine: evidence that amphetamine releases dopamine from noradrenergic neurons in the medial prefrontal cortex. Neurochem Res.

[B70] Stephans SE, Yamamoto BY (1995). Effect of repeated methamphetamine administrations on dopamine and glutamate efflux in rat prefrontal cortex. Brain Res.

[B71] Behrens MM, Ali SS, Dao DN, Lucero J, Shekhtman G, Quick KL, Dugan LL (2007). Ketamine-induced loss of phenotype of fast-spiking interneurons is mediated by NADPH-oxidase. Science.

[B72] Kinney JW, Davis CN, Tabarean I, Conti B, Bartfai T, Behrens MM (2006). A specific role for NR2A-containing NMDA receptors in the maintenance of parvalbumin and GAD67 immunoreactivity in cultured interneurons. J Neurosci.

[B73] Castner SA, Vosler PS, Goldman-Rakic PS (2005). Amphetamine sensitization impairs cognition and reduces dopamine turnover in primate prefrontal cortex. Biol Psychiatry.

[B74] Pierce RC, Kalivas PW (1997). A circuitry model of the expression of behavioral sensitization to amphetamine-like psychostimulants. Brain Res Rev.

[B75] Abi-Dargham A, Mawlawi O, Lombardo I, Gil R, Martinez D, Huang Y, Hwang D-R, Keilp J, Kochan L, Heertum RV, Gorman JM, Laruelle M (2002). Prefrontal dopamine D1 receptors and working memory in schizophrenia. J Neurosci.

[B76] Laruelle M (2000). The role of endogenous sensitization in the pathophysiology of schizophrenia: Implications from recent brain imaging studies. Brain Res Rev.

[B77] Lieberman JA, Sheitman BB, Kinon BJ (1997). Neurochemical Sensitization in the Pathophysiology of Schizophrenia: Deficits and Dysfunction in Neuronal Regulation and Plasticity. Neuropsychopharmacology.

[B78] Arnsten AFT, Goldman-Rakic PS (1998). Noise stress impairs prefrontal cortical cognitive function in monkeys. Arch Gen Psychiatry.

[B79] Hutson PH, Patel S, Jay MT, Barton CL (2004). Stress-induced increase of cortical dopamine metabolism: attenuation by a tachykinin NK1 receptor antagonist. Eur J Pharmacol.

[B80] Deutch AY, Lee MC, Gillham MH, Cameron DA, Goldstein M, Iadarola MJ (1991). Stress selectively increases fos protein in dopamine neurons innervating the prefrontal cortex. Cereb Cortex.

[B81] Murphy BL, Arnsten AFT, Goldman-Rakic PS, Roth RH (1996). Increased dopamine turnover in the prefrontal cortex impairs spatial working memory performance in rats and monkeys. Proc Natl Acad Sci USA.

[B82] Murphy BL, Arnsten AFT, Jentsch JD, Roth RH (1996). Dopamine and spatial working memory in rats and monkeys: pharmocaological reversal of stress-induced impairment. J Neurosci.

[B83] Mizoguchi K, Yuzurihara M, Ishige A, Sasaki H, Chui DH, Tabira T (2000). Chronic stress induces impairment of spatial working memory because of prefrontal dopaminergic dysfunction. J Neurosci.

[B84] Qin P, Xu H, Laursen TM, Vestergaard M, Mortensen PB (2005). Risk for schizophrenia and schizophrenia-like psychosis among patients with epilepsy: population based cohort study. BMJ.

[B85] Sachdev P (1998). Schizophrenia-like psychosis and epilepsy: the status of the association. Am J Psychiatry.

[B86] Toone BK (2000). The psychoses of epilepsy. J Neurol Neurosurg Psychiatry.

[B87] Adachi N, Matsuura M, Hara T, Oana Y, Okubo Y, Kato M, Onuma T (2002). Psychoses and epilepsy: are interictal and postictal psychoses distinct clinical entities?. Epilepsia.

[B88] Kanemoto K, Tsuji T, Kawasaki J (2001). Reexamination of interictal psychoses based on DSM IV psychosis classification and international epilepsy classification. Epilepsia.

[B89] Avoli M, Louvel J, Pumain R, Kohling R (2005). Cellular and molecular mechanisms of epilepsy in the human brain. Prog Neurobiol.

[B90] Fisher RS, van Emde Boas W, Blume W, Elger C, Genton P, Lee P, Engel J (2005). Epileptic seizures and epilepsy: definitions proposed by the International League Against Epilepsy (ILAE) and the International Bureau for Epilepsy (IBE). Epilepsia.

[B91] Bowser DN, Wagner DA, Czajkowski C, Cromer BA, Parker MW, Wallace RH, Harkin LA, Mulley JC, Marini C, Berkovic SF, Williams DA, Jones MV, Petrou S (2002). Altered kinetics and benzodiazepine sensitivity of a GABAA receptor subunit mutation [gamma 2(R43Q)] found in human epilepsy. Proc Natl Acad Sci USA.

[B92] Loup F, Wieser HG, Yonekawa Y, Aguzzi A, Fritschy JM (2000). Selective alterations in GABAA receptor subtypes in human temporal lobe epilepsy. J Neurosci.

[B93] Ando N, Morimoto K, Watanabe T, Ninomiya T, Suwaki H (2004). Enhancement of central dopaminergic activity in the kainate model of temporal lobe epilepsy: implication for the mechanism of epileptic psychosis. Neuropsychopharmacology.

[B94] Starr MS (1996). The role of dopamine in epilepsy. Synapse.

[B95] Bandyopadhyay S, Gonzalez-Islas C, Hablitz JJ (2005). Dopamine enhances spatiotemporal spread of activity in rat prefrontal cortex. J Neurophysiol.

[B96] Braff DL, Geyer MA, Swerdlow NR (2001). Human studies of prepulse inhibition of startle: normal subjects, patient groups, and pharmacological studies. Psychopharmacology (Berl).

[B97] Braver TS, Barch DM, Cohen JD (1999). Cognition and control in schizophrenia: a computational model of dopamine and prefrontal function. Biol Psychiatry.

[B98] Geyer MA, Krebs-Thomson K, Braff DL, Swerdlow NR (2001). Pharmacological studies of prepulse inhibition models of sensorimotor gating deficits in schizophrenia: a decade in review. Psychopharmacology (Berl).

[B99] Swerdlow NR, Shoemaker JM, Kuczenski R, Bongiovanni MJ, Neary AC, Tochen LS, Saint Marie RL (2006). Forebrain D1 function and sensorimotor gating in rats: effects of D1 blockade, frontal lesions and dopamine denervation. Neurosci Lett.

[B100] Braff DL, Freedman R, Kenneth L Davis, Dennis Charney, Joseph T Coyle, Charles Nemeroff (2002). Endophenotypes in studies of the genetics of schizophrenia. Neuropsychopharmacology: The Fifth Generation of Progress.

[B101] Swerdlow NR, Geyer MA, Braff DL (2001). Neural circuit regulation of prepulse inhibition of startle in the rat: current knowledge and future challenges. Psychopharmacology (Berl).

[B102] Tenn CC, Fletcher PJ, Kapur S (2003). Amphetamine-sensitized animals show a sensorimotor gating and neurochemical abnormality similar to that of schizophrenia. Schizophr Res.

[B103] Wang M, Vijayraghavan S, Goldman-Rakic PS (2004). Selective D2 receptor actions on the functional circuitry of working memory. Science.

[B104] Seeman P, Weinshenker D, Quirion R, Srivastava LK, Bhardwaj SK, Grandy DK, Premont RT, Sotnikova TD, Boksa P, El-Ghundi M, O'dowd BF, George SR, Perreault ML, Mannisto PT, Robinson S, Palmiter RD, Tallerico T (2005). Dopamine supersensitivity correlates with D2High states, implying many paths to psychosis. Proc Natl Acad Sci USA.

[B105] Seeman P, Schwarz J, Chen JF, Szechtman H, Perreault M, McKnight GS, Roder JC, Quirion R, Boksa P, Srivastava LK, Yanai K, Weinshenker D, Sumiyoshi T (2006). Psychosis pathways converge via D2High dopamine receptors. Synapse.

[B106] Yamashita K, Tanaka S (2005). Parametric study of dopaminergic neuromodulatory effects in a reduced model of the prefrontal cortex. Neurocomputing.

[B107] Durstewitz D, Seamans JK, Sejnowski TJ (2000). Dopamine-mediated stabilization of delay-period activity in a network model of prefrontal cortex. J Neurophysiol.

[B108] Howard A, Tamas G, Soltesz I (2005). Lighting the chandelier: new vistas for axo-axonic cells. Trends Neurosci.

[B109] Wang XJ, Tegner J, Constantinidis C, Goldman-Rakic PS (2004). Division of labor among distinct subtypes of inhibitory neurons in a cortical microcircuit of working memory. Proc Natl Acad Sci USA.

[B110] Seamans JK, Yang CR (2004). The principal features and mechanisms of dopamine modulation in the prefrontal cortex. Prog Neurobiol.

[B111] Yang CR, Seamans JK, Gorelova N (1999). Developing a neuronal model for the pathophysiology of schizophrenia based on the nature of electrophysiological actions of dopamine in the prefrontal cortex. Neuropsychopharmacology.

[B112] Gorelova N, Seamans JK, Yang CR (2002). Mechanisms of dopamine activation of fast-spiking interneurons that exert inhibition in rat prefrontal cortex. J Neurophysiol.

